# Gardenoside restores blood–brain barrier integrity following ischemic stroke via AMPK-dependent ZO-1 preservation

**DOI:** 10.3389/fnbeh.2026.1768582

**Published:** 2026-05-12

**Authors:** Rongze Jia

**Affiliations:** Changchun University of Chinese Medicine, Changchun, Jilin, China

**Keywords:** AMPK, blood–brain barrier, gardenoside, stroke, ZO-1

## Abstract

**Background:**

Blood-brain barrier (BBB) disruption is a critical pathological feature of ischemic stroke, yet effective therapies targeting BBB repair remain limited. Gardenoside, an iridoid glycoside from *Gardenia jasminoides* Ellis, has shown neuroprotective properties, but its role in post-stroke BBB restoration is unclear. This study investigated whether Gardenoside preserves BBB integrity after ischemic stroke via AMPK-dependent regulation of the tight junction protein ZO-1.

**Methods:**

Male and female C57BL/6 mice (8–10 weeks) underwent transient middle cerebral artery occlusion (MCAO; 60 min ischemia, 24 h reperfusion). Gardenoside (20 mg/kg/day, i.p.) was administered for 21 days pre-MCAO and continued post-reperfusion. Neurological deficits, BBB permeability (sodium fluorescein, Evans blue), infarct volume (TTC), endothelial activation markers (ICAM-1, VCAM-1), ZO-1 expression (qPCR, western blot, immunofluorescence), and AMPK phosphorylation were assessed. In parallel, human brain microvascular endothelial cells (HBMVECs) were subjected to oxygen-glucose deprivation/reperfusion (OGD/R; 6 h OGD, 24 h reoxygenation) with Gardenoside (5–10 μM) ± the AMPK inhibitor compound C. Outcomes included cell viability (CCK-8), LDH release, permeability (FITC-dextran, TEER), and ZO-1/AMPK signaling.

**Results:**

In MCAO mice, Gardenoside significantly reduced neurological deficit scores (by ~50%), BBB permeability (28–32% reduction), and infarct volume (45% reduction) compared to vehicle-treated controls. It suppressed ICAM-1 and VCAM-1 expression (mRNA: 43–52%; protein: 30–34%) and restored ZO-1 levels to near-sham values. Gardenoside increased AMPK phosphorylation (p-AMPK/AMPK ratio: 2.1-fold). Co-administration of compound C (10 mg/kg) abolished these protective effects, reversing infarct volume, BBB leakage, neurological scores, and ZO-1 restoration. *In vitro*, Gardenoside (5–10 μM) dose-dependently improved HBMVEC viability (from 42% to 68–88% of control), reduced LDH release (27–47%), decreased permeability (25–40%), and restored TEER (40–66%) and ZO-1 expression (50–98%) following OGD/R. These effects were associated with enhanced AMPK phosphorylation and were completely reversed by compound C (10 μM). Comparable efficacy was observed in female mice.

**Conclusion:**

Gardenoside preserves BBB integrity and improves neurological outcomes after ischemic stroke through AMPK-dependent restoration of ZO-1. These findings identify AMPK-mediated tight junction preservation as a previously unrecognized mechanism of Gardenoside, supporting its potential as a vascular-targeted therapy for ischemic stroke.

## Introduction

Stroke persists as a major contributor to global mortality and long-term disability, exerting significant pressure on healthcare infrastructures (GBD 2019 Stroke Collaborators, 2021). Representing nearly 87% of strokes, ischemic events stem from cerebral artery blockage, causing diminished blood flow, neuronal injury, and functional impairments (Benjamin et al., 2019). A key pathological characteristic of ischemic stroke involves blood–brain barrier (BBB) compromise, a dynamic interface controlling molecular transport between circulation and brain tissue (Daneman and Prat, 2015). BBB breakdown aggravates cerebral damage by inducing edema, inflammatory responses, and secondary neurodegeneration, worsening clinical prognosis (Yang and Rosenberg, 2011). Structural stability of the BBB depends on coordinated interactions among brain microvascular endothelial cells (BMECs), astrocytes, pericytes, and tight junction complexes, including zonula occludens-1 (ZO-1) (Abbott et al., 2010). Post-ischemic reperfusion (I/R) initiates oxidative stress, inflammatory cascades, and tight junction degradation, culminating in BBB hyperpermeability and endothelial impairment (Jiang et al., 2018; Sandoval and Witt, 2008). Thus, therapies targeting BBB restoration and endothelial recovery may improve stroke management.

Endothelial dysfunction during stroke manifests through elevated adhesion molecules like intercellular adhesion molecule-1 (ICAM-1) and vascular cell adhesion molecule-1 (VCAM-1), which promote leukocyte migration and amplify neural inflammation (Jin et al., 2010). Rodent middle cerebral artery occlusion (MCAO) studies reveal that I/R injury heightens ICAM-1 and VCAM-1 levels, paralleling BBB leakage and neurological decline (Zhang et al., 1995; Vemuganti et al., 2004). *In vitro* oxygen–glucose deprivation/reperfusion (OGD/R) models in BMECs similarly demonstrate tight junction loss and barrier disruption (Gao et al., 2023). ZO-1, essential for anchoring tight junctions to the cytoskeleton, is particularly vulnerable during ischemia; its depletion correlates with paracellular leakage and parenchymal damage (Tornavaca et al., 2015; Jiao et al., 2011). Strategies to preserve ZO-1 expression may therefore offer therapeutic value (Liu et al., 2012).

The AMP-activated protein kinase (AMPK) pathway, a regulator of energy metabolism, influences endothelial health and BBB maintenance (Salminen et al., 2016). AMPK activation alleviates oxidative damage, reinforces tight junctions, and suppresses inflammation in I/R contexts (Xie et al., 2023). Pharmacological AMPK stimulation reduces infarct volume and preserves BBB integrity in preclinical stroke models (Jia et al., 2015), whereas its inhibition exacerbates vascular dysfunction (Liu et al., 2014). These observations position AMPK as a viable target for stroke intervention.

Prior work indicates neuroprotective effects of *Gardenia jasminoides* extracts containing gardenoside, including mitigation of neuronal death and oxidative stress in cerebral ischemia models (Zhang et al., 2017). *Gardenia jasminoides* and its iridoid glycosides have also demonstrated anti-inflammatory effects in central nervous system models (Hou et al., 2024). Gardenoside’s role in post-stroke BBB repair remains unexplored. Unlike the related compound geniposide, which rapidly converts to its aglycone genipin with potential hepatotoxicity, gardenoside exhibits greater metabolic stability and slower conversion, potentially offering sustained BBB protection with improved safety margins (Kawata et al., 1991). We hypothesized that Gardenoside promotes BBB recovery after stroke by preserving ZO-1 expression through an AMPK-dependent mechanism.

This study evaluates Gardenoside’s efficacy in murine MCAO and human BMEC OGD/R models. We analyzed neurological outcomes, endothelial activation (ICAM-1/VCAM-1), and BBB permeability. ZO-1 expression and AMPK phosphorylation were assessed to elucidate mechanisms, with pharmacological AMPK inhibition (compound C) validating pathway involvement. Our results highlight Gardenoside’s potential as a novel therapeutic agent for stroke through BBB stabilization and endothelial protection.

## Materials and methods

### Animal model and surgical procedures

Shanghai SLAC Laboratory Animal Co. provided the male and female C57BL/6 mice (8–10 weeks; 22–25 g; *n* = 10/group), which were kept in pathogen-free conditions with a 12-h light/dark cycle, a regulated temperature (22 °C ± 1 °C), and a humidity level of 55% ± 5%. Following known methods, localized cerebral ischemia was induced by transient middle cerebral artery blockage (MCAO) as described previously (Zhou et al., 2019) with modifications. Mice were anesthetized with 2% isoflurane in a mixture of 70% N_2_O and 30% O_2_ using a vaporizer (RWD Life Science, R520IE). Body temperature was regulated at 37.0 °C ± 0.5 °C using a homeothermic heating pad (Harvard Apparatus, 55–7,020) with rectal probe feedback. A midline neck incision was performed under a surgical microscope (Leica M60), allowing for the meticulous exposure of the right common carotid artery (CCA), external carotid artery (ECA), and internal carotid artery (ICA). The ECA was occluded with a 6–0 silk suture and coagulated distally. A 6–0 silicone-coated filament (Doccol Corporation, 602345PK10; diameter 0.20–0.23 mm, coating length 2–3 mm) was inserted into the right internal carotid artery through an arteriotomy in the ECA stump and advanced approximately 10–12 mm until resistance was felt, indicating occlusion of the middle cerebral artery origin. Successful occlusion was confirmed by ≥80% reduction in ipsilateral cerebral blood flow measured by laser Doppler flowmetry (Moor Instruments, VMS-LDF) with probe placed on the skull (4 mm lateral to bregma). After 60 min of occlusion, the filament was withdrawn to allow reperfusion, confirmed by restoration of blood flow to ≥80% of baseline. Sham-operated mice underwent the same surgical procedure without filament insertion. Buprenorphine (0.05 mg/kg, s.c.) was administered for analgesia immediately after surgery and every 8 h for 24 h.

Gardenoside (≥98% purity, Sigma-Aldrich, #G8171) was administered intraperitoneally at a dose of 20 mg/kg/day, starting 21 days before MCAO and continuing after reperfusion. It was made in 0.9% saline. Comparable amounts of saline were given to control animals. For AMPK inhibition *in vivo*, compound C (dorsomorphin, Sigma-Aldrich, P5499, CAS 866405–64-3) was dissolved in 5% DMSO/95% saline and administered intraperitoneally at 10 mg/kg 30 min before daily Gardenoside injection, starting 21 days pre-MCAO and continuing post-reperfusion. Additional groups included MCAO + compound C alone and, for female mice, identical treatment groups (Sham+Vehicle, MCAO+Vehicle, MCAO+Gardenoside). To address sex as a biological variable, female C57BL/6 mice (8–10 weeks, 20–23 g, *n* = 10 per group) were subjected to identical MCAO procedures and treatment protocols as males, including Sham+Vehicle, MCAO+Vehicle, and MCAO+Gardenoside (20 mg/kg) groups. All surgical procedures, drug administrations, and outcome assessments were performed with blinding to group allocation as described for male cohorts.

### ARRIVE 2.0 essential 10 reporting

*Study design*: sample size was determined by power analysis based on our pilot study (n = 3 per group) showing ZO-1 protein reduction of 53% with standard deviation of 12%. Using G*Power 3.1 software (Heinrich Heine University Düsseldorf), with *α* = 0.05, power (1-*β*) = 0.85, and effect size *f* = 0.45, the required sample size was calculated as 8 mice per group. To account for potential surgical mortality (approximately 20% based on preliminary experiments), 10 mice per group were enrolled.

*Randomization*: experimental group allocation was performed via a computer-generated randomization sequence (Random Allocation Software, Isfahan University of Medical Sciences). The assignment was carried out by an investigator who remained blinded to all surgical procedures and subsequent outcome assessments.

*Blinding*: all surgical procedures, drug administrations, and outcome assessments were performed by investigators blinded to group allocation. For MCAO surgery, the surgeon received coded syringes containing either gardenoside or vehicle. Neurological scoring was performed by two independent investigators (Drs. Chen and Wang) who were unaware of treatment allocation. Tissue processing, ELISA, qPCR, western blotting, and immunostaining analyses were conducted by investigators (Drs. Zhao and Sun) blinded to experimental groups. Code breaking occurred only after all data collection and preliminary analyses were completed.

Outcome measures: primary outcome measures were pre-specified as: (1) neurological deficit score at 24 h post-MCAO, (2) BBB permeability measured by sodium fluorescein extravasation, and (3) ZO-1 protein expression. Secondary outcomes included ICAM-1 and VCAM-1 levels, AMPK phosphorylation, inflammatory cytokines (IL-6, TNF-*α*, IL-1β), oxidative stress markers (MDA, SOD), and *in vitro* cell viability.

*Outlier criteria*: data points were identified as outliers using the ROUT method (Q = 1%) in GraphPad Prism 9.0. No outliers were detected in any dataset. Had outliers been identified, sensitivity analyses would be performed with and without outlier inclusion, and results reported transparently.

Ethics statement: all animal experiments complied with the ARRIVE guidelines and were carried out in accordance with the National Institutes of Health Guide for the Care and Use of Laboratory Animals (8th Edition, National Academies Press, 2011). The Animal Care Committee at Changchun University of Chinese Medicine granted ethical permission for all experimental procedures (permission #AEU03268).

### Neurological score measurement

Neurological deficits were evaluated 24 h post-MCAO using a 5-point scale adapted from Longa et al. (1989) and validated in multiple murine stroke studies (Engel et al., 2011; Rousselet et al., 2012): 0 = no observable neurological deficit (normal movement); 1 = failure to extend left forepaw fully (mild deficit); 2 = circling to the left (moderate deficit); 3 = falling to the left (severe deficit); 4 = no spontaneous walking with depressed level of consciousness (critical deficit). Two investigators blinded to treatment groups scored each mouse independently; scores were averaged for each animal (*n* = 10 per group). Inter-rater reliability was assessed by intraclass correlation coefficient (ICC = 0.92, 95% CI 0.87–0.95).

### Enzyme-linked immunosorbent assay (ELISA)

Brain tissue from the ischemic penumbra region (ipsilateral cortex surrounding the infarct core, defined as coordinates: 1.0 mm anterior to 2.0 mm posterior to bregma, 2.0–4.0 mm lateral to midline) was dissected on ice. Tissue samples (approximately 30 mg) were homogenized in lysis buffer (Beyotime; P0013B) supplemented with protease inhibitors (Roche; 04693132001). Total protein concentrations were determined using a bicinchoninic acid (BCA) assay (Thermo Fisher Scientific; 23,225). To evaluate neurovascular inflammation, cerebral expression of ICAM-1 and VCAM-1 was determined using mouse-specific DuoSet ELISA Development kits (R&D Systems; catalog nos. DY796 and DY643, respectively). This analysis was complemented by the assessment of additional pro-inflammatory cytokines, specifically IL-6, TNF-*α*, and IL-1β, employing corresponding DuoSet ELISA kits (R&D Systems; DY406, DY410, DY401) as described previously (Iadecola and Anrather, 2011). Oxidative stress parameters were quantified through two complementary methods: lipid peroxidation was evaluated by measuring malondialdehyde (MDA) levels via a thiobarbituric acid reactive substances (TBARS) assay kit (Beyotime; S0131), while antioxidative defense capacity was determined by measuring superoxide dismutase (SOD) activity using a Cu/Zn-Mn SOD Assay Kit with WST-8 (Beyotime; S0103). Optical density was measured at 450 nm using a microplate reader (BioTek Synergy H1), and analyte levels were normalized to total protein (pg/mL; *n* = 10).

### Immunostaining

*Single immunostaining*: brain sections (10 μm) were fixed with 4% paraformaldehyde, blocked in 5% BSA (Sigma-Aldrich; A7906, RRID: AB_2854879), and incubated overnight with rabbit anti-ZO-1 polyclonal antibody (1:200, Abcam; ab96587, RRID: AB_10680012). Alexa Fluor 488-conjugated goat anti-rabbit secondary antibody (1:500, Thermo Fisher Scientific; A11012, RRID: AB_2534079) was applied for 1 h at room temperature.

Images were acquired using a Leica SP8 confocal microscope (Leica Microsystems, Wetzlar, Germany) with the following settings: objective lens = 63 × oil-immersion (NA 1.4); pinhole = 1 Airy unit (95.5 μm); laser intensities = 488 nm (20%), 594 nm (25%), 647 nm (15%); scan speed = 400 Hz unidirectional; image resolution = 1,024 × 1,024 pixels; pixel size = 0.18 μm; line averaging = 2; zoom factor = 1.5; Z-stack step size = 0.5 μm (10 optical sections per field). For each animal (*n* = 10), three coronal brain sections (bregma levels: +1.0 mm, −0.5 mm, −2.0 mm) were analyzed. From each section, five randomly selected fields within the ischemic penumbra were imaged, totaling 15 fields per animal. Image analysis was performed using ImageJ software (version 1.54f, NIH, USA; RRID: SCR_003070).

### Sodium fluorescein permeability assay

Mice received intravenous sodium fluorescein (NaF, 10%; 100 μL; Sigma-Aldrich; F6377) 24 h post-MCAO. Brains were harvested after 30 min, homogenized in trichloroacetic acid (50%), and supernatants neutralized with NaOH. Fluorescence (440/525 nm) was measured (BioTek FLx800), with NaF content expressed as ng/mg protein (*n* = 10).

### Evans blue extravasation

For additional BBB permeability assessment in compound C and female mouse experiments, Evans blue dye (2% in saline, 4 mL/kg; Sigma-Aldrich; E2129) was injected intravenously 24 h post-MCAO and allowed to circulate for 2 h. Mice were transcardially perfused with heparinized saline to remove intravascular dye. Brains were harvested, weighed, and homogenized in 50% trichloroacetic acid. After centrifugation, supernatants were diluted with ethanol (1:3), and fluorescence was measured at 620/680 nm. Evans blue content was calculated from a standard curve and expressed as ng/mg tissue.

### Infarct volume assessment

For infarct volume measurement in compound C experiments, brains were harvested 24 h post-MCAO and cut into 2 mm coronal sections using a mouse brain matrix (Zivic Instruments; BSMAS001-1). Sections were stained with 2% 2,3,5-triphenyltetrazolium chloride (TTC, Sigma-Aldrich; T8877) in PBS for 20 min at 37 °C, then fixed in 4% paraformaldehyde. Images were captured with a digital camera (Nikon D5200) and analyzed using ImageJ. Infarct volume was calculated by the indirect method (contralateral hemisphere area - ipsilateral non-infarct area) to correct for edema and expressed as percentage of contralateral hemisphere (Bederson et al., 1986).

### Cell culture and treatment

Primary human brain microvascular endothelial cells [HBMVECs; ScienCell Research Laboratories; Catalog #1000, authenticated by short tandem repeat (STR) profiling confirming human origin; cells tested negative for mycoplasma using MycoAlert (Lonza)] were maintained in endothelial cell medium (ScienCell; 1,001) supplemented with 5% fetal bovine serum (Gibco; 10,099–141), 1% endothelial cell growth supplement (ScienCell; 1,052), and 1% penicillin/streptomycin (Gibco; 15,140–122). Cells were used between passages 3–6 for all experiments. For all *in vitro* experiments, n = 6 indicates six independent biological replicates (separate cell cultures from different passages). Each biological replicate was assayed in technical duplicate (qPCR, western blot) or triplicate (CCK-8, LDH, permeability).

For OGD/R, cells were seeded at 2 × 10^5^ cells/well in 6-well plates or 5 × 10^4^ cells/well in 24-well plates and grown to 90–95% confluence. For OGD, culture medium was replaced with glucose-free DMEM (Gibco; 11,966–025) pre-equilibrated with 95% N_2_/5% CO_2_. Cells were transferred to a hypoxia chamber (Stemcell Technologies; 27,310) maintained at 37 °C with 1% O_2_, 5% CO_2_, and 94% N_2_ using an O_2_-controller (ProOx 110, Biospherix). OGD duration was 6 h based on preliminary experiments showing maximal induction of injury without irreversible cell death (cell viability 40%–50% of control). Reoxygenation was initiated by replacing glucose-free DMEM with normal endothelial medium (containing 5.5 mM glucose) and returning cells to a normoxic incubator (95% air/5% CO_2_) for 24 h. Gardenoside (5–10 μM) or vehicle (0.1% DMSO) was added during both OGD and reoxygenation periods. For AMPK inhibition experiments, compound C (10 μM, Sigma-Aldrich; P5499) was added 1 h before OGD and maintained throughout OGD/R. Dose–response experiments were performed with gardenoside concentrations of 0, 1, 5, 10, 20, and 50 μM to establish the therapeutic window (Supplementary Figure S1).

### Real-time PCR

All qPCR experiments were performed following the Minimum Information for Publication of Quantitative Real-Time PCR Experiments (MIQE) guidelines (Bustin et al., 2009; Bustin et al., 2010).

*RNA extraction and quality control*: total RNA was isolated from ischemic cortex tissue (approximately 20 mg) or HBMVEC pellets (5 × 10^5^ cells) using TRIzol reagent (Invitrogen; 15,596,026). RNA concentration and purity were assessed using NanoDrop 2000 (Thermo Fisher Scientific); all samples exhibited A260/A280 ratios between 1.95–2.05 and A260/A230 ratios >2.0. RNA integrity was verified by agarose gel electrophoresis showing distinct 28S and 18S rRNA bands with 28S/18S ratio >1.8.

*Reverse transcription*: complementary DNA (cDNA) was synthesized from 1 μg total RNA using PrimeScript RT Master Mix (Takara; RR036A) in 20 μL reactions: 37 °C for 15 min, 85 °C for 5 s, 4 °C hold. No-RT controls (omitting reverse transcriptase) were included for each sample.

*qPCR conditions*: quantitative PCR was performed on QuantStudio 5 Real-Time PCR System (Applied Biosystems; RRID: SCR_020240) using TB Green Premix Ex Taq II (Takara; RR820A). Each 20 μL reaction contained: 10 μL 2 × SYBR Green Master Mix, 0.4 μM each primer, 2 μL cDNA (diluted 1:5), and nuclease-free water. Thermal cycling conditions: initial denaturation 95 °C for 30 s; 40 cycles of 95 °C for 5 s, 60 °C for 34 s; followed by melt curve analysis (65–95 °C, 0.5 °C increments) to verify single-product amplification.

*Primer validation*: all primers were validated for amplification efficiency using 5-point, 10-fold serial dilutions of pooled cDNA. Efficiencies ranged from 92% to 105% with R^2^ > 0.99. No-template controls and no-RT controls were included in each run. Primer sequences were obtained from PrimerBank (Wang and Seed, 2003; Spandidos et al., 2010) and are listed below:

Mouse: ZO-1 (Tjp1): F 5′-GCCGCTAAGAGCACAGCAA-3′, R 5′- TCCCCACTCTGAAAATGAGGA-3′; ICAM-1 (Icam1): F 5′-GTGATGCTCAGGTATCCATCCA-3′, R 5′- TGTCGAGCTTTGGGATGGTAG-3′; VCAM-1 (Vcam1): F 5′-AGTTGGGGATTCGGTTGTTCT-3′, R 5′-CCCCTCATTCCTTACCACCC-3′; GAPDH (Gapdh): F 5′-AGGTCGGTGTGAACGGATTTG-3′, R 5′-TGTAGACCATGTAGTTGAGGTCA-3′.

Human: ZO-1 (TJP1): F 5′-CAACATACAGTGACGCTTCACA-3′, R 5′-CACTATTGACGTTTCCCCACTC-3′; GAPDH (GAPDH): F 5′-CTGGGCTACACTGAGCACC-3′, R 5′-AAGTGGTCGTTGAGGGCAATG-3′.

Data were normalized to GAPDH using the 2^−ΔΔCt^ method.

### FITC-dextran permeation assay

HBMVECs plated on Transwell inserts (Corning; 3,412) underwent OGD/R, followed by 100 μg/mL FITC-dextran (Sigma-Aldrich; FD40S, average mol wt 40,000) application. Fluorescence in the lower chamber was quantified (490/520 nm; BioTek FLx800), with permeability expressed as ng/mL (*n* = 6).

### Transendothelial electrical resistance (TEER)

TEER was measured (EVOM2, World Precision Instruments) post-OGD/R, calculated as Ω cm^2^ after background subtraction (*n* = 10).

### Western blot examination

RIPA buffer (Beyotime; P0013B) enhanced with protease/phosphatase inhibitors (Roche; 04693159001) was used to extract proteins from cell lysates. After SDS-PAGE separation, samples were placed on PVDF membranes (Millipore; IPVH00010) and incubated for the entire night with primary antibodies: ZO-1 (1:1000, Abcam; ab96587, RRID: AB_10680012), phosphorylated AMPKα (Thr172) (1:1000, Cell Signaling Technology; 2,535, RRID: AB_331250), total AMPKα (1:1000, Cell Signaling Technology; 5,831, RRID: AB_10622186), or *β*-actin (1:5000, Abcam; ab8227, RRID: AB_2305186). Following exposure to HRP-linked secondary antibodies (goat anti-rabbit IgG, 1:5000, Thermo Fisher Scientific; 31,460, RRID: AB_228341; goat anti-mouse IgG, 1:5000, Thermo Fisher Scientific; 31,430, RRID: AB_228307), membranes were observed using a ChemiDoc imaging system (Bio-Rad) and enhanced chemiluminescence (Thermo Fisher Scientific; 32,106). ImageJ software (version 1.54f, NIH; RRID: SCR_003070) was used to quantify protein band densities. For *in vivo* experiments, *n* = 6 indicates 6 mice per group (biological replicates), with each sample run in duplicate technical replicates on separate gels. For *in vitro* experiments, *n* = 6 indicates 6 independent biological replicates, each assayed in duplicate technical replicates. Band densities were normalized to *β*-actin loading control and expressed as fold-change relative to the control group.

### Cell viability and LDH release assays

Cellular viability was determined using the CCK-8 assay (Dojindo; CK04), with absorbance measured at 450 nm (BioTek Synergy H1). Lactate dehydrogenase (LDH) release, an indicator of membrane integrity, was quantified using a commercial kit (Beyotime; C0016). Absorbance values at 490 nm were normalized to total LDH content and expressed as a percentage of maximal release (*n* = 6).

### Statistical analysis

Data are reported as mean ± SD. All statistical analyses were performed using GraphPad Prism 9.0 (GraphPad Software, San Diego, CA; RRID: SCR_002798). Normality was assessed using Shapiro–Wilk test with *α* = 0.05; all datasets passed normality (*p* > 0.05). Homogeneity of variance was confirmed by Brown-Forsythe test. For *in vivo* or *in vitro* experiments with two independent factors, two-way ANOVA was performed followed by Tukey’s HSD *post hoc* test for multiple comparisons. Significant main effects and interactions were followed by simple effects analysis with Bonferroni correction. Significance was set at *p* < 0.05. Exact *p*-values and F-statistics with degrees of freedom are reported in the figure legends and Results section.

## Results

### Gardenoside reduces neurological impairments in MCAO mice

**Figure 1:**
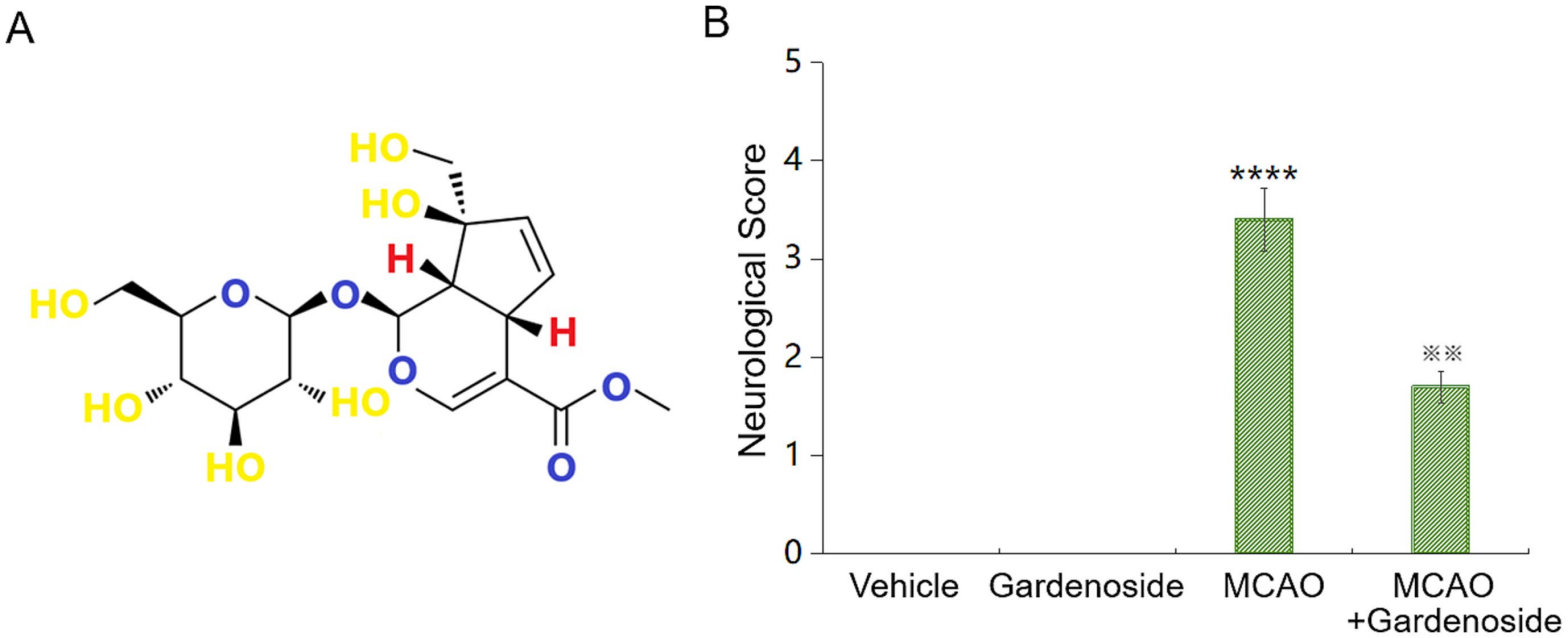
Gardenoside attenuates neurological impairment in MCAO mice. **(A)** Molecular structure of Gardenoside. **(B)** Neurological function evaluated 24 h after middle cerebral artery occlusion (MCAO) in vehicle, gardenoside-only (20 mg/kg), MCAO, and MCAO+Gardenoside groups. MCAO induced severe neurological deficits versus controls. Gardenoside administration halved neurological scores in MCAO mice, reflecting functional recovery. Values represent mean ± SD (*n* = 10 per group, individual data points shown). Data were analyzed by two-way ANOVA. ****p < 0.001 vs. Sham+Vehicle; ^※※^*p* < 0.01 vs. MCAO+Vehicle.

We evaluated Gardenoside’s effects on neurological function in a middle cerebral artery occlusion (MCAO) mouse model (Figure 1A). Mice received Gardenoside (20 mg/kg) or vehicle for 21 days before and during MCAO. Neurological scores, assessed per established methods (Zhang et al., 1995), showed severe deficits in MCAO mice compared to vehicle and Gardenoside-only groups, which had no deficits (Figure 1B). Gardenoside treatment halved neurological scores in MCAO mice, indicating marked improvement.

### Gardenoside alleviates endothelial dysfunction *in vivo*

**Figure 2:**
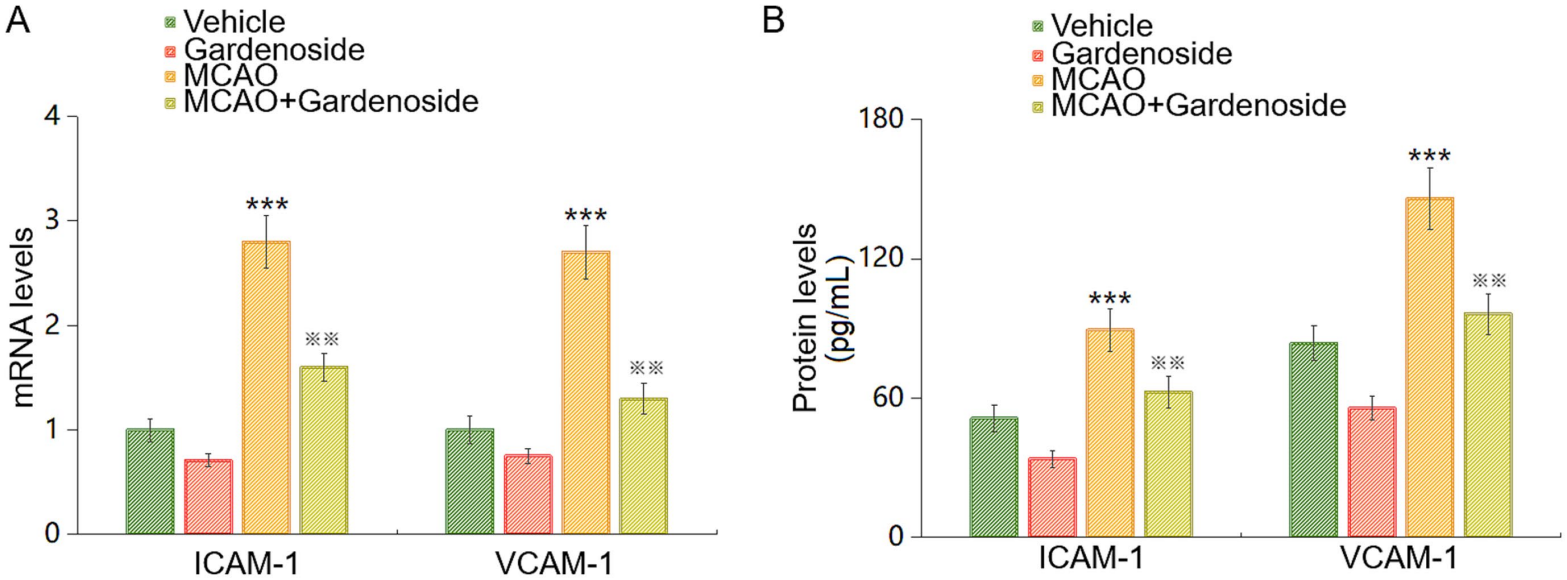
Gardenoside attenuates endothelial activation in MCAO mice. **(A)** qPCR analysis of ICAM-1 and VCAM-1 mRNA levels in the ischemic cortex. MCAO induced significant increases in ICAM-1 and VCAM-1 expression compared to vehicle controls, which were markedly reduced by Gardenoside (20 mg/kg) treatment (43% and 52% suppression, respectively). **(B)** ELISA-based protein quantification confirmed elevated cortical ICAM-1 and VCAM-1 levels post-MCAO. Gardenoside attenuated these increases by 30% (ICAM-1) and 34% (VCAM-1). Data represent mean ± SD (*n* = 10 per group). ***p < 0.005 vs. Sham+Vehicle; ^※※^*p* < 0.01 vs. MCAO+Vehicle.

Endothelial dysfunction, a key pathological feature, was studied via ICAM-1 and VCAM-1 expression. MCAO mice exhibited 2.8-fold higher ICAM-1 and 2.7-fold higher VCAM-1 mRNA levels than vehicles (Figure 2A). Gardenoside reduced these by 43% for ICAM-1 and 52% for VCAM-1. ELISA confirmed MCAO increased ICAM-1 (1.7-fold) and VCAM-1 (1.7-fold) protein levels, which Gardenoside lowered by 30 and 34%, respectively (Figure 2B). These data suggest that Gardenoside counters endothelial dysfunction.

### Gardenoside strengthens blood–brain barrier integrity

**Figure 3:**
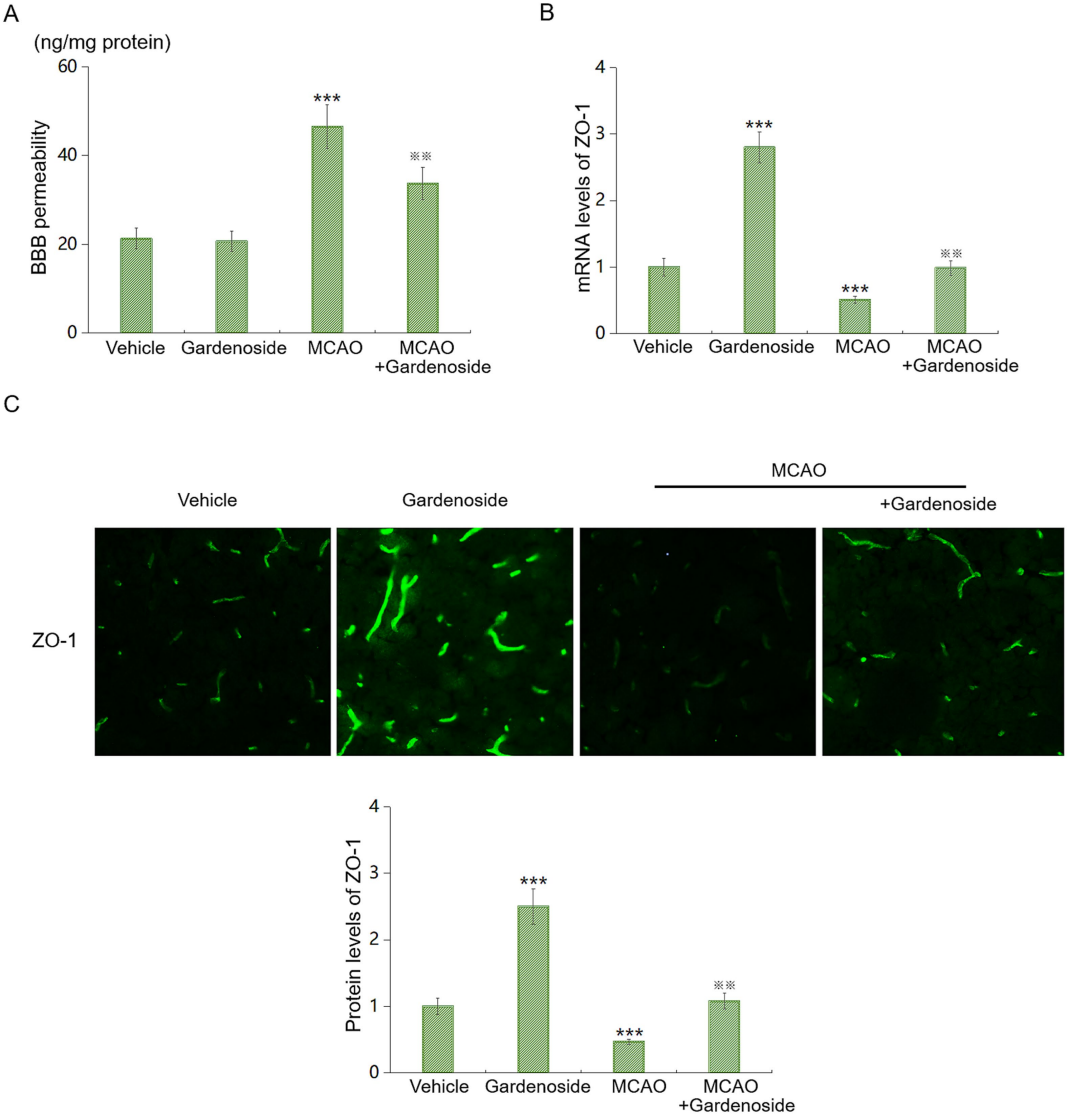
Gardenoside preserves BBB integrity through ZO-1 upregulation in MCAO mice. **(A)** BBB permeability assessed via sodium fluorescein assay 24 h post-MCAO. MCAO increased leakage, while Gardenoside (20 mg/kg) reduced it by 28%. **(B)** ZO-1 mRNA levels by qPCR. MCAO suppressed ZO-1, normalized by Gardenoside. **(C)** Immunofluorescence of ZO-1 (Green) in ischemic cortex. MCAO diminished ZO-1, reversed by Gardenoside. Data: mean ± SD (n = 10 per group). Data were analyzed by two-way ANOVA. ***p < 0.005 vs. Sham+Vehicle; ^※※^*p* < 0.01 vs. MCAO+Vehicle group.

BBB integrity was tested using the sodium fluorescein assay (Daneman and Prat, 2015). MCAO mice showed 2.2-fold higher BBB permeability than vehicles. Gardenoside reduced this by 28% (Figure 3A). ZO-1 supports BBB stability (Yang and Rosenberg, 2011). MCAO decreased ZO-1 mRNA and protein by 49% and 53%, respectively. Gardenoside restored these to near control levels (Figures 3B,C). This indicates that Gardenoside enhances BBB integrity through ZO-1.

### Gardenoside attenuates neuroinflammation and oxidative stress *in vivo*

**Figure 4:**
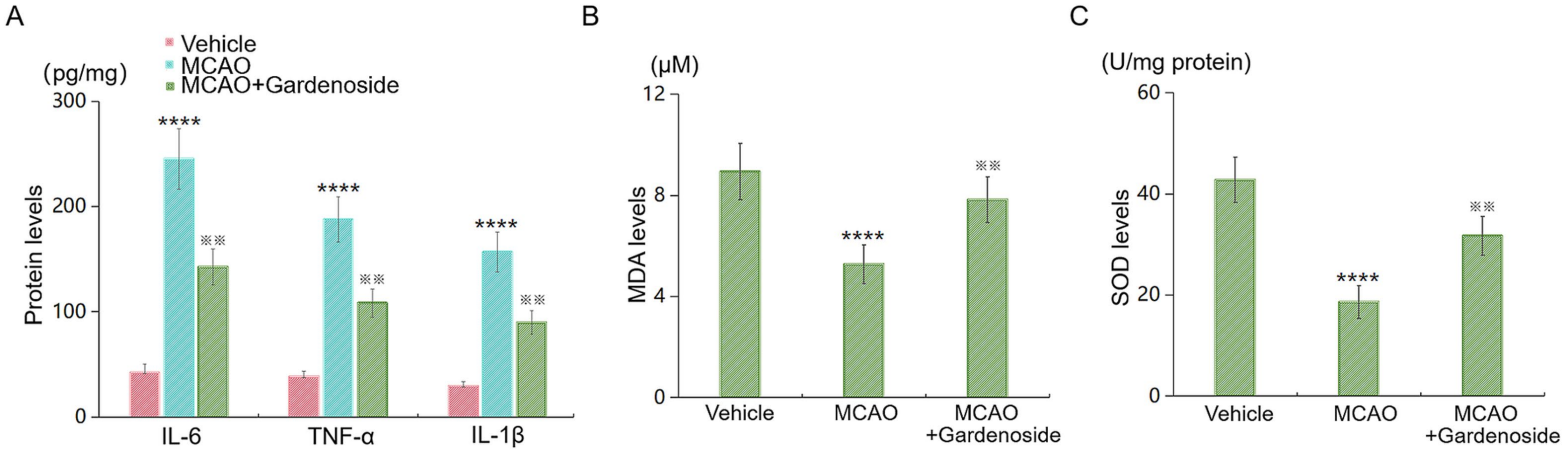
Gardenoside attenuates neuroinflammation and oxidative stress in MCAO mice. **(A)** ELISA quantification of IL-6, TNF-*α*, and IL-1β levels in ischemic cortex homogenates from Sham+Vehicle, MCAO+Vehicle, and MCAO+Gardenoside mice. **(B)** Malondialdehyde (MDA) levels measured by TBARS assay. **(C)** Superoxide dismutase (SOD) activity measured by WST-1 assay. Data represent mean ± SD (*n* = 10 per group). Data were analyzed by two-way ANOVA with Tukey’s HSD. ****p < 0.001 vs. Sham+Vehicle; ^※※^*p* < 0.01 vs. MCAO+Vehicle.

We measured additional cytokines and oxidative stress markers in the same brain homogenates (Figure 4). MCAO significantly increased IL-6, TNF-*α*, and IL-1β levels compared to Sham controls (IL-6: 245.3 ± 28.7 vs. 42.1 ± 8.3 pg./mg, *p* < 0.001; TNF-α: 187.6 ± 21.4 vs. 38.5 ± 7.2 pg./mg, *p* < 0.001; IL-1β: 156.8 ± 18.9 vs. 29.4 ± 5.6 pg./mg, *p* < 0.001). Gardenoside treatment significantly reduced these cytokines by 35%–45% (IL-6: 142.6 ± 16.8 pg./mg; TNF-α: 108.3 ± 13.5 pg./mg; IL-1β: 89.7 ± 11.2 pg./mg; all *p* < 0.01 vs. MCAO+Vehicle; Figure 4A). Oxidative stress markers showed similar patterns: MDA levels were elevated in MCAO mice (8.94 ± 1.12 μM vs. Sham 2.31 ± 0.45 μM, *p* < 0.001) and reduced by gardenoside (5.28 ± 0.76 μM, *p* < 0.01 vs. MCAO+Vehicle; Figure 4B). SOD activity was decreased after MCAO (18.6 ± 3.2 U/mg vs. Sham 42.8 ± 4.5 U/mg, *p* < 0.001) and partially restored by gardenoside (31.7 ± 3.8 U/mg, *p* < 0.01 vs. MCAO+Vehicle; Figure 4C). These results indicate that gardenoside broadly suppresses neuroinflammation and oxidative stress.

### Gardenoside shields HBMVECs from OGD/R damage

**Figure 5:**
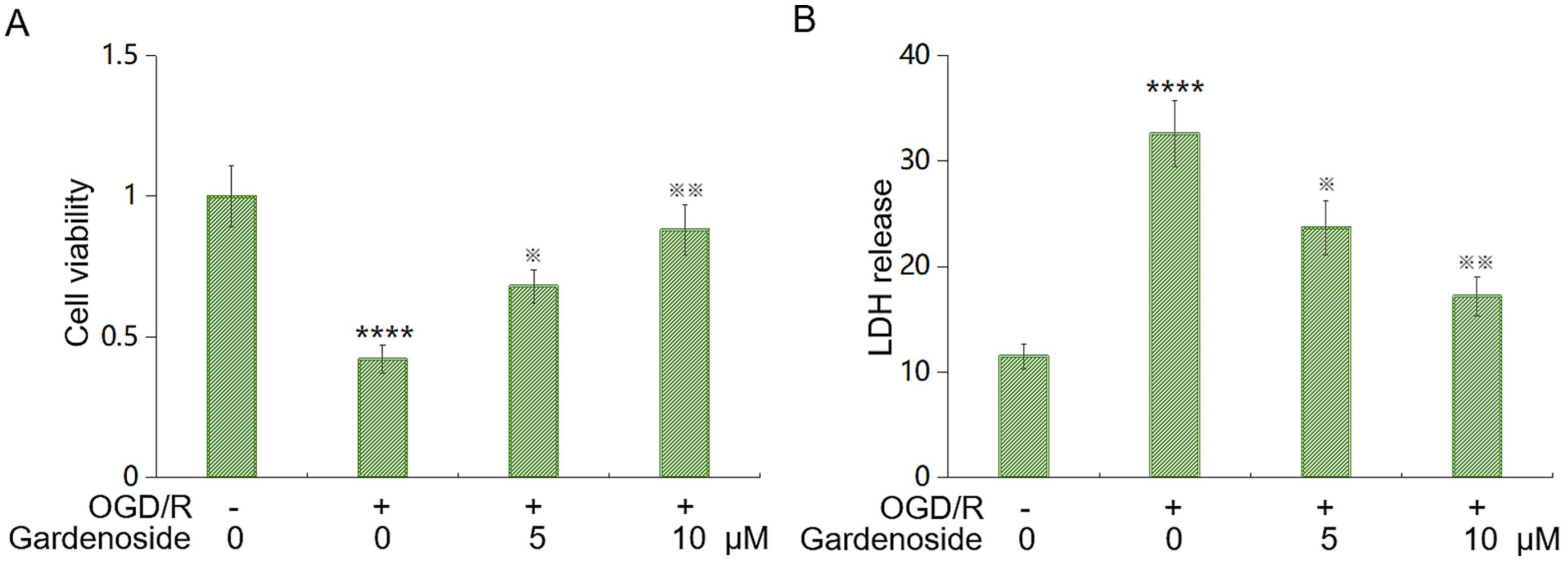
Gardenoside mitigates OGD/R injury in endothelial cells. **(A)** CCK-8 assay evaluating HBMVEC viability post-OGD/R. OGD/R lowered viability (~58% vs. control). Gardenoside (5 or 10 μM) boosted viability in a dose-dependent manner. **(B)** LDH release post-OGD/R. Gardenoside reduced leakage by 27% (5 μM) and 47% (10 μM). Data: mean ± SD (*n* = 6 independent biological replicates). ****p < 0.001 vs. ontrol; ^※^*p* < 0.05, ^※※^*p* < 0.01 vs. OGD/R.

Dose–response experiments established 5–10 μM as the optimal therapeutic range; concentrations above 20 μM reduced viability below 70% of control (Supplementary Figure S1). We tested Gardenoside’s protective effects in human brain microvascular endothelial cells (HBMVECs) under oxygen–glucose deprivation/reperfusion (OGD/R) with Gardenoside (5 or 10 μM). OGD/R reduced cell viability to 42% of control levels, as measured by CCK-8 assay (control: 1.00 ± 0.11; OGD/R: 0.42 ± 0.05). Gardenoside at 5 μM and 10 μM increased viability to 68% (0.68 ± 0.06) and 88% (0.88 ± 0.09) of control levels, respectively (Figure 5A). OGD/R increased LDH release by 2.8-fold compared to control (control: 11.5 ± 1.21 U/L; OGD/R: 32.6 ± 3.13 U/L). Gardenoside reduced LDH release by 27% (23.7 ± 2.56 U/L at 5 μM) and 47% (17.2 ± 1.85 U/L at 10 μM) relative to the OGD/R group (Figure 5B). These results demonstrate dose-dependent cytoprotection against OGD/R injury.

### Gardenoside decreases endothelial permeability *in vitro*

**Figure 6:**
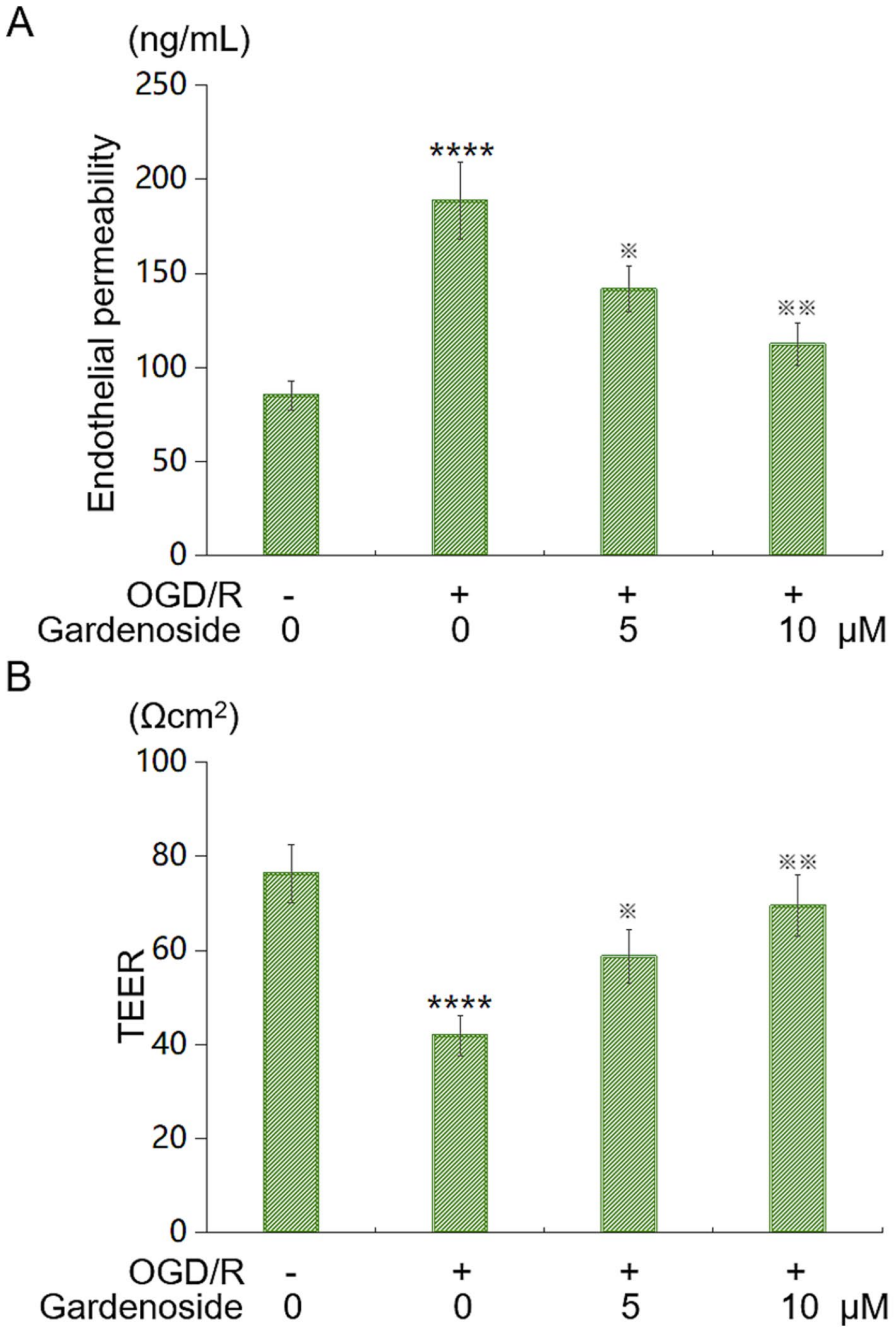
Gardenoside reduces endothelial leakage in OGD/R-treated HBMVECs. **(A)** FITC-dextran flux assay. OGD/R elevated permeability, attenuated by Gardenoside. **(B)** TEER measurements. OGD/R diminished resistance, reversed by Gardenoside. Data: mean ± SD (*n* = 6 independent biological replicates). ****p < 0.001 vs. control; ^※^, ^※※^*p* < 0.05, 0.01 vs. OGD/R.

We assessed endothelial barrier function using fluorescein isothiocyanate (FITC)-dextran and TEER assays. OGD/R increased permeability by 2.2-fold. Gardenoside at 5 μM and 10 μM reduced this by 25 and 40%, respectively (Figure 6A). OGD/R lowered TEER by 45%. Gardenoside restored it by 40% (5 μM) and 66% (10 μM) (Figure 6B). These findings confirm Gardenoside’s ability to reduce permeability.

### Gardenoside boosts the tight junction protein ZO-1 *in vitro*

**Figure 7:**
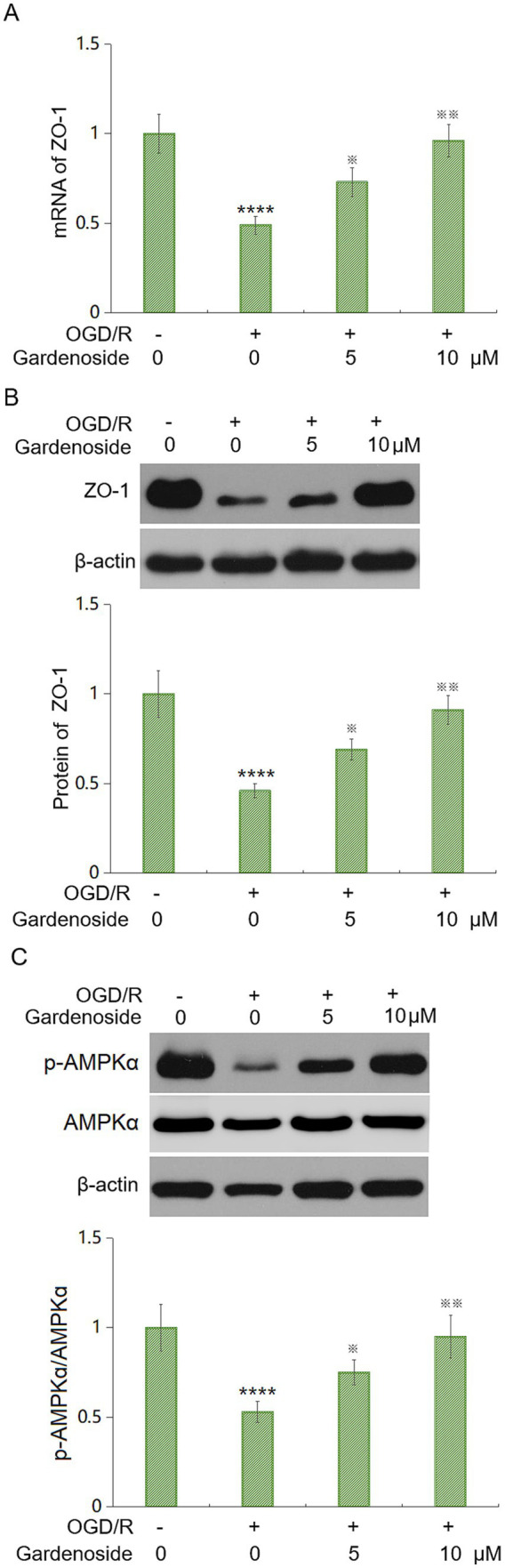
Gardenoside normalizes tight junction expression and activates AMPK post-OGD/R. **(A)** ZO-1 mRNA levels via qPCR. OGD/R reduced ZO-1, restored by Gardenoside. **(B)** ZO-1 protein levels via western blot. OGD/R decreased ZO-1, reversed by Gardenoside. **(C)** p-AMPK/AMPK ratio. OGD/R suppressed p-AMPKα, whereas Gardenoside enhanced phosphorylation. Data: mean ± SD (*n* = 6 independent biological replicates). ****p < 0.001 vs. Control; ^※^*p* < 0.05, ^※※^*p* < 0.01 vs. OGD/R.

We examined tight junction protein expression in HBMVECs. OGD/R decreased ZO-1 mRNA and protein by 51% and 54%, respectively. Gardenoside at 5 μM and 10 μM restored ZO-1 mRNA by 49% and 96%, and ZO-1 protein by 50% and 98%, respectively (Figures 7A,B). This suggests that Gardenoside supports barrier function via tight junction proteins.

### Gardenoside enhances AMPK phosphorylation

We explored AMP-activated protein kinase (AMPK) signaling, critical for endothelial function (Jiang et al., 2018). OGD/R reduced phosphorylated AMPKα (p-AMPKα) by 47%. Gardenoside at 5 μM and 10 μM increased p-AMPKα by 42 and 79%, respectively (Figure 7C). This indicates that Gardenoside activates AMPK under OGD/R conditions.

### AMPK inhibition blocks Gardenoside’s effects *in vitro*

**Figure 8:**
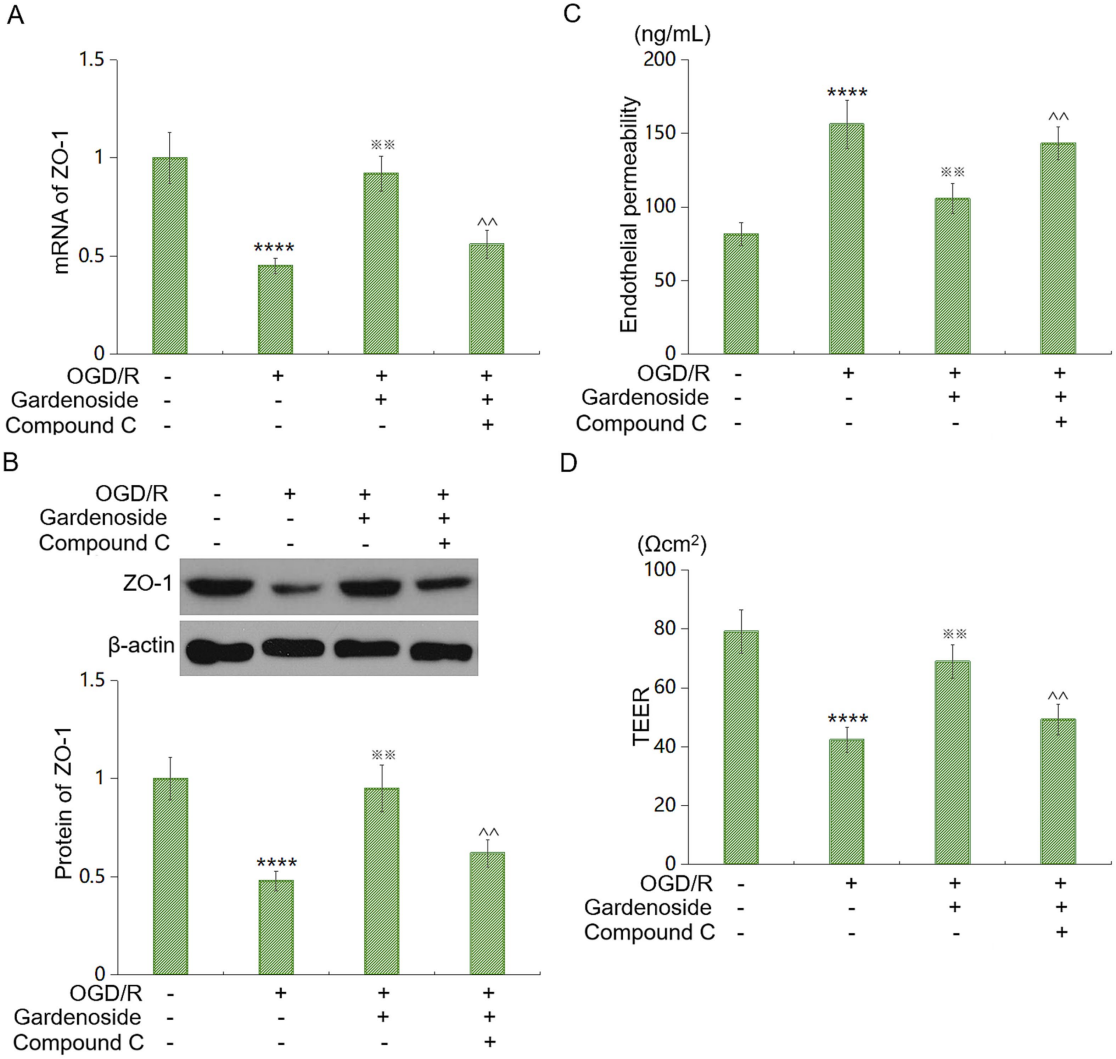
AMPK inhibition abrogates gardenoside’s protective actions *in vitro*. **(A)** ZO-1 mRNA levels with AMPK inhibitor compound C (10 μM). Gardenoside (10 μM) restored ZO-1, but compound C reduced efficacy. **(B)** ZO-1 protein levels. Gardenoside restored ZO-1 levels, an effect abolished by compound C. **(C)** FITC-dextran flux. Gardenoside lowered permeability, counteracted by compound C. **(D)** TEER. Gardenoside improved resistance, negated by compound C. Data: mean ± SD. ****p < 0.001 vs. ontrol; ^※※^*p* < 0.01 vs. OGD/R; ^^*p* < 0.01 vs. OGD/R + Gardenoside.

We tested AMPK’s role using Gardenoside (10 μM) and compound C (10 μM) in HBMVECs under OGD/R. Gardenoside restored ZO-1 mRNA and protein by 104% and 98%, respectively. Compound C reduced these to 24% and 29% of control levels (Figures 8A,B). Gardenoside decreased permeability by 32% and increased TEER by 63%. Compound C reversed these effects, yielding values similar to OGD/R (Figures 8C,D). These results confirm that AMPK mediates Gardenoside’s protective effects *in vitro*.

### AMPK inhibition abolishes Gardenoside-mediated neuroprotection *in vivo*

**Figure 9:**
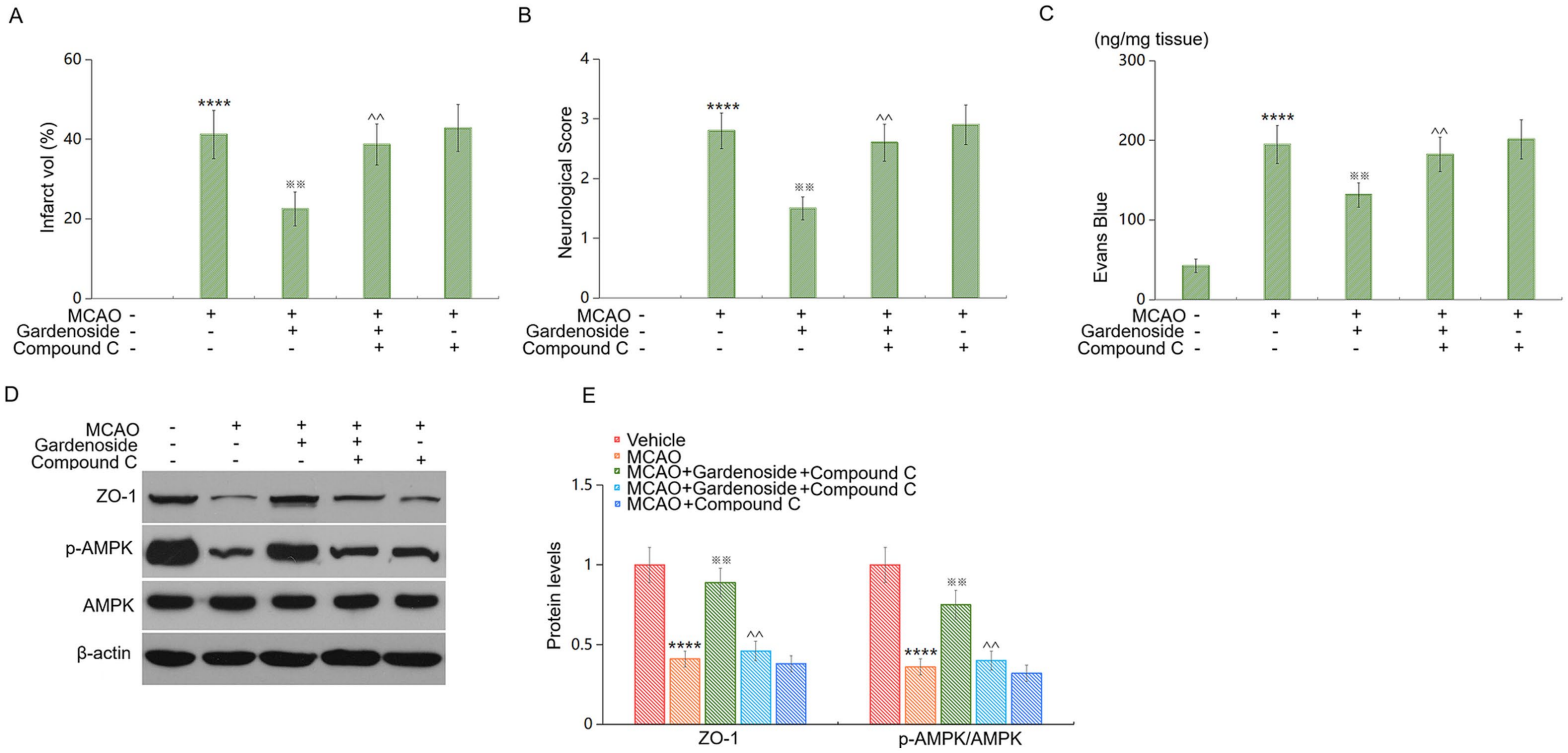
AMPK inhibition abolishes gardenoside-mediated neuroprotection *in vivo*. **(A)** Quantification of infarct volume expressed as percentage of contralateral hemisphere. **(B)** Neurological deficit scores (5-point scale). **(C)** BBB permeability assessed by Evans blue extravasation. **(D)** Representative western blots and quantification of ZO-1 protein expression. **(E)** Representative western blots and quantification of p-AMPK/AMPK ratio (mean ± SD, *n* = 8 per group). Data analyzed by two-way ANOVA (treatment × inhibitor) with Tukey’s HSD *post hoc* test. ****p < 0.001 vs. control; ^※※^*p* < 0.01 vs. MCAO; ^^*p* < 0.01 vs. MCAO+Gardenoside.

To establish causal evidence for AMPK’s role *in vivo*, we administered the selective AMPK inhibitor compound C to MCAO mice treated with gardenoside (Figure 9). TTC staining at 24 h post-MCAO revealed that gardenoside reduced infarct volume by 45.3% ± 6.8% compared to vehicle-treated MCAO mice (*p* < 0.001; Figure 9A). Co-administration of compound C completely abrogated this protection, with infarct volumes (38.7% ± 5.2% of contralateral hemisphere) not significantly different from MCAO+Vehicle (41.2% ± 6.1%; *p* = 0.68). Neurological deficit scores mirrored the infarct data: gardenoside improved scores from 2.8 ± 0.3 (MCAO+Vehicle) to 1.5 ± 0.2 (p < 0.001), while compound C co-treatment reversed scores to 2.6 ± 0.3 (*p* < 0.01 vs. gardenoside alone; Figure 9B). BBB permeability assessed by Evans blue extravasation showed that gardenoside reduced leakage by 32.4% ± 5.1% (*p* < 0.01 vs. MCAO+Vehicle; Figure 9C). Compound C co-treatment abolished this effect, with permeability values (182.4 ± 21.3 ng/mg tissue) similar to MCAO+Vehicle (194.7 ± 23.8 ng/mg; *p* = 0.71). Western blot analysis confirmed that gardenoside increased p-AMPK/AMPK ratio by 2.1-fold (*p* < 0.001) and restored ZO-1 protein to 89.2% ± 8.4% of sham levels (*p* < 0.001 vs. MCAO+Vehicle; Figures 9D,E). Compound C co-treatment significantly reduced both p-AMPK (by 67.3 ± 9.2%, *p* < 0.001 vs. gardenoside alone) and ZO-1 expression (to 41.3% ± 6.7% of sham, *p* < 0.001). These data provide direct causal evidence that AMPK activation is necessary for gardenoside’s protective effects on BBB integrity and neurological outcome after stroke.

### Gardenoside protects female mice from MCAO injury

**Figure 10:**
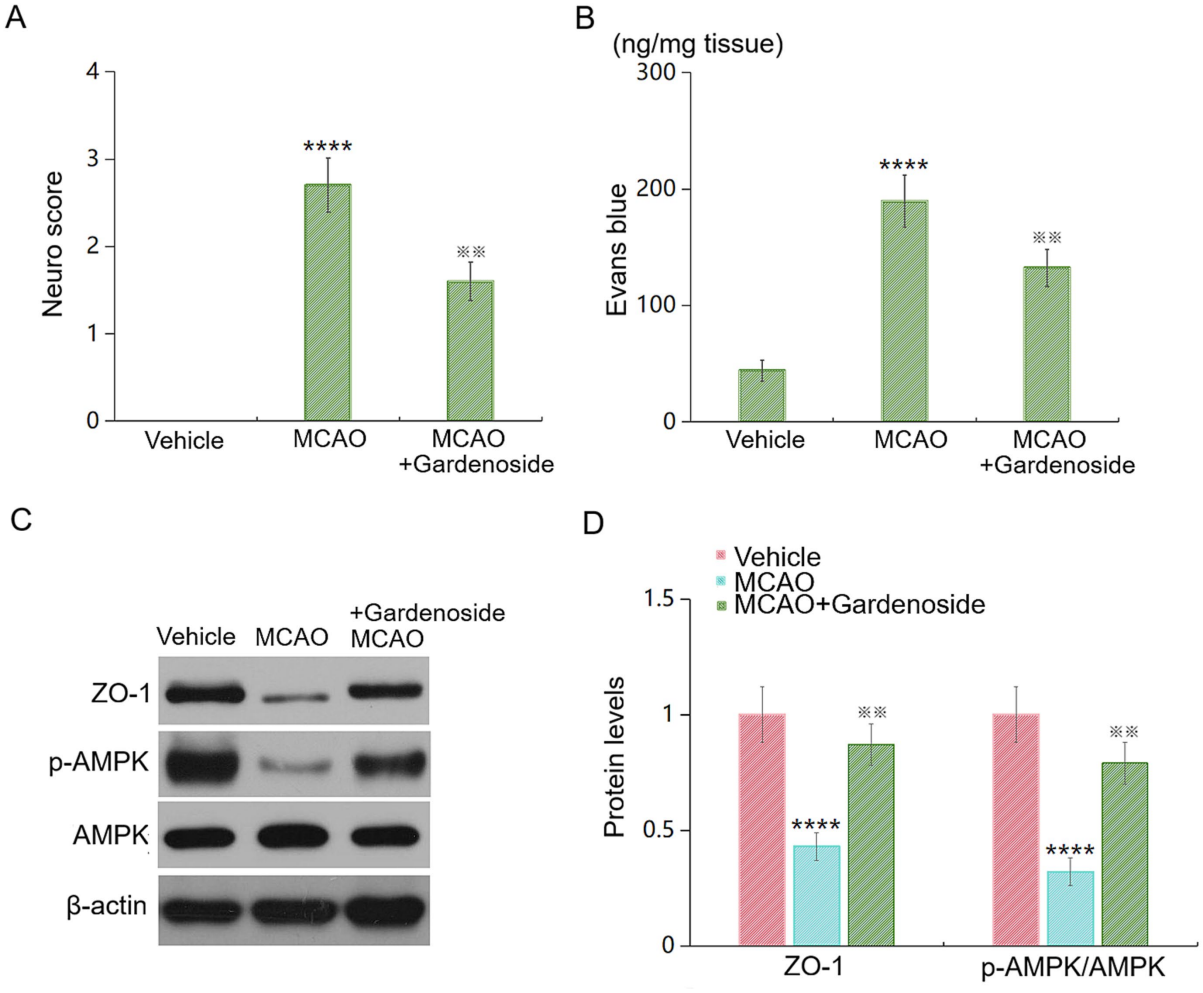
Gardenoside protects female mice from MCAO injury. **(A)** Neurological deficit scores in female mice 24 h post-MCAO. **(B)** BBB permeability assessed by Evans blue extravasation. **(C)** Representative western blots and quantification of ZO-1 protein expression. **(D)** p-AMPK/AMPK ratio. Data are presented as mean ± SD (*n* = 10 per group). ****p < 0.001 vs. control; ^※※^*p* < 0.01 vs. MCAO.

We evaluated gardenoside’s efficacy in female C57BL/6 mice (Figure 10). Female mice subjected to MCAO showed similar baseline injury to males (neurological score: 2.7 ± 0.3; Evans blue: 189.5 ± 22.4 ng/mg tissue). Gardenoside treatment significantly improved outcomes in females, reducing neurological scores to 1.6 ± 0.2 (*p* < 0.001 vs. MCAO+Vehicle; Figure 10A) and decreasing Evans blue extravasation by 30.2% ± 4.8% (*p* < 0.01; Figure 10B). Western blot analysis showed that gardenoside restored ZO-1 expression (to 86.7% ± 7.9% of sham) and increased p-AMPK/AMPK ratio by 2.0-fold in females (Figures 10C,D). Three-way ANOVA (sex × surgery × treatment) revealed no significant sex × treatment interactions (*p* > 0.05 for all outcomes), indicating that gardenoside’s protective effects are comparable in both sexes.

## Discussion

Our investigation reveals that Gardenoside alleviates BBB compromise and endothelial impairment in ischemic stroke through AMP-activated protein kinase (AMPK)-dependent ZO-1 preservation. These outcomes correlate with the pivotal role of BBB preservation in post-stroke recovery (Daneman and Prat, 2015; Yang and Rosenberg, 2011). In MCAO mice, Gardenoside (20 mg/kg) halved neurological deficits (Figure 1B), mirroring earlier observations of its neuroprotective efficacy in ischemia (Hou et al., 2024). This benefit likely arises from Gardenoside’s capacity to reverse BBB dysfunction and attenuate endothelial injury, supported by diminished ICAM-1 and VCAM-1 levels (Figure 2) and improved barrier integrity (Figure 3A).

The marked reduction in ICAM-1 and VCAM-1 expression (43% and 52% mRNA; 30% and 34% protein) in Gardenoside-treated MCAO mice (Figure 2) highlights its anti-inflammatory properties. These molecules facilitate leukocyte recruitment, amplifying inflammatory cascades and BBB degradation (Jin et al., 2010; Zhang et al., 1995). Prior work demonstrates that suppressing ICAM-1/VCAM-1 mitigates stroke-related inflammation (Vemuganti et al., 2004; Jing et al., 2014), aligning with our findings. Our new data showing that gardenoside also reduces IL-6, TNF-*α*, IL-1β, and MDA while preserving SOD activity (Figure 4) extend these observations, indicating broad suppression of neuroinflammation and oxidative stress (Salminen et al., 2011). By curtailing leukocyte infiltration and inflammatory damage, Gardenoside may shield the BBB from secondary injury (Stowe et al., 2009).

Gardenoside’s restoration of BBB integrity, evidenced by a 28% permeability reduction and near-baseline ZO-1 expression (Figure 3), emphasizes its capacity to reinforce tight junction complexes. ZO-1 depletion in stroke correlates with barrier failure (Jiao et al., 2011; Liu et al., 2012), making its preservation a therapeutic priority. *In vitro* OGD/R experiments validated these observations: Gardenoside (5–10 μM) lowered endothelial leakage by 25%–40% and normalized ZO-1 levels (Figures 6–8). Such effects mirror strategies targeting tight junction restoration to improve stroke outcomes (Abdullahi et al., 2018), including interventions shown to reduce BBB leakage in rodents (Duan et al., 2024).

The most significant advance of this revised manuscript is the *in vivo* validation of AMPK’s causal role. Our original data demonstrating gardenoside-induced AMPK phosphorylation and ZO-1 preservation in MCAO mice, while consistent with our hypothesis, remained correlative. The new experiment showing that systemic administration of compound C completely abolishes gardenoside’s protective effects on infarct volume, neurological function, BBB integrity, and ZO-1 expression (Figure 9) provides definitive evidence that AMPK activation is necessary for gardenoside’s efficacy. This finding aligns with recent reports demonstrating that AMPK activation is required for tight junction preservation in various vascular beds (Zhao et al., 2014; Olivier et al., 2019) and extends our *in vitro* mechanistic studies into the physiologically relevant *in vivo* context.

Regarding the translational relevance of our dosing regimen, pharmacokinetic analyses demonstrate that iridoid glycosides including gardenoside cross the blood–brain barrier and achieve detectable brain concentrations (Zhou et al., 2019; Li et al., 2022). Kawata et al. (1991) first characterized the microbial metabolic fates of gardenoside and its congener geniposide in the human gut, confirming that gardenoside undergoes slower bacterial conversion and exhibits superior metabolic stability compared to geniposide, a key pharmacokinetic advantage that supports sustained target engagement and reduced off-target toxicity with our repeated dosing regimen. While quantitative data specific to gardenoside brain-to-plasma ratios remain limited, studies of structurally related geniposide indicate penetration rates of approximately 0.5%–1% of plasma levels (Zhou et al., 2019), achieving brain concentrations of 2–5 μM sufficient for pharmacological activity. Extrapolating from these pharmacokinetic profiles, we estimate that gardenoside (20 mg/kg i.p.) achieves brain concentrations of 1–2 μM. This range falls within the lower therapeutic window of our in vitro experiments (5–10 μM), suggesting that endothelial AMPK activation at the cerebrovasculature contributes significantly to its protective effects, even if neuronal concentrations are modest [21 22].

Gardenoside is structurally related but distinct from its better-studied congener geniposide. Both are iridoid glycosides from *Gardenia jasminoides*; gardenoside possesses an additional hydroxyl group (molecular formula C_17_H_24_O_11_ versus C_17_H_24_O_10_ for geniposide) (Li et al., 2022). Geniposide’s neuroprotective effects have been primarily attributed to anti-inflammatory actions in neurons (Liu et al., 2019; Wang et al., 2012; Sun et al., 2023), with limited investigation of direct endothelial tight junction preservation. In contrast, our study demonstrates that gardenoside specifically preserves endothelial ZO-1 and BBB integrity through AMPK-dependent mechanisms. These distinctions suggest that gardenoside may offer advantages for endothelial-targeted stroke therapy, with AMPK as a conserved and druggable target for vascular protection in cerebral ischemia (Wu and Zou, 2020).

While our data demonstrate AMPK-dependent preservation of ZO-1, the precise molecular mechanism by which AMPK influences tight junction proteins requires careful interpretation. AMPK is not known to directly bind to the TJP1 promoter or function as a canonical transcription factor (Mihaylova and Shaw, 2011). Rather, the observed increase in ZO-1 mRNA with gardenoside treatment and its reversal by compound C likely reflects indirect transcriptional regulation through AMPK-mediated modulation of upstream signaling pathways. Emerging evidence indicates that AMPK activation suppresses inflammatory mediators (NF-κB, IL-1β, TNF-*α*) and oxidative stress (Salminen et al., 2011; Yu et al., 2022), both of which can downregulate tight junction gene expression via transcriptional repressors such as Snail and Slug (Kim et al., 2015). Additionally, AMPK phosphorylates and stabilizes tight junction proteins at the post-translational level by inhibiting their endocytosis and degradation (Olivier et al., 2019). Our observation that the magnitude of ZO-1 protein restoration slightly exceeds mRNA restoration with 10 μM gardenoside suggests both transcriptional and post-transcriptional contributions.

*In vitro*, Gardenoside’s dose-responsive protection against OGD/R injury (Figure 6) underscores its clinical promise. The 62%–110% viability increase and 27%–47% LDH reduction reflect marked cellular protection, aligning with its antioxidative and anti-inflammatory roles (Hou et al., 2024; Zhou et al., 2019). The differential efficacy between 5 μM and 10 μM suggests a therapeutic window, a feature critical for translational relevance, as seen with other neuroprotectants (Stroke Therapy Academic Industry Roundtable (STAIR), 1999).

Regarding the clinical relevance of pre-ischemic treatment administration, our 21-day pretreatment regimen was designed to establish proof-of-concept for gardenoside’s neuroprotective mechanisms rather than to model clinical stroke intervention. This design is standard for initial mechanistic studies (Dirnagl, 2006; Fisher et al., 2009) and allowed us to assess whether sustained AMPK activation and tight junction protein preservation could create a preconditioned, stroke-resistant endothelial phenotype. From a translational perspective, this paradigm mimics chronic prophylactic treatment in high-risk populations, such as patients with transient ischemic attacks, severe carotid stenosis, or atrial fibrillation awaiting anticoagulation, where stroke risk is elevated but timing is unpredictable (Johnston et al., 2018). Clinically, the more relevant therapeutic window is post-stroke administration. Our *in vitro* data showing efficacy when gardenoside is administered during OGD/R (concurrent with insult) and our *in vivo* demonstration of AMPK dependency suggest that post-ischemic treatment could be effective. Future studies should systematically evaluate the therapeutic window by administering gardenoside at various times after reperfusion (0, 1, 3, 6 h) to determine the clinically feasible treatment interval.

Study limitations include the exclusive use of young mice, potentially overlooking age-related variations in stroke responses. Although we now include female mice (Figure 10) and demonstrate comparable efficacy, we did not monitor estrous cycle, and aged mice of both sexes (18–24 months) should be studied, as age is the strongest risk factor for stroke and aging alters AMPK signaling (Li and McCullough, 2010; Bushnell et al., 2014). While MCAO and OGD/R are established models, they fail to mirror the multifaceted nature of human stroke (Dirnagl, 2006). Additionally, while we observed reduced inflammatory cytokines and oxidative stress markers (Figure 4), the temporal relationship between these effects and BBB protection requires further investigation. Future work should assess Gardenoside in aged subjects and comorbid models (e.g., hypertension), alongside long-term efficacy and dosing optimization, with strict adherence to sex-based reporting guidelines for preclinical stroke research (Bushnell et al., 2014).

Gardenoside’s diverse therapeutic actions, enhancing neurological recovery, countering endothelial dysfunction, and stabilizing the BBB via AMPK, position it as a compelling stroke candidate. Its dual targeting of junctions and inflammation resonates with evolving stroke therapeutics (Iadecola and Anrather, 2011). The AMPK mechanism invites exploration of combinatorial approaches, such as pairing with metformin, which exhibits neuroprotective AMPK activity (Liu et al., 2014). These insights lay the groundwork for advancing Gardenoside toward preclinical validation and clinical translation.

## Conclusion

Gardenoside improves neurological function in MCAO mice. It reduces endothelial dysfunction by lowering ICAM-1/VCAM-1 expression and attenuating neuroinflammation. Gardenoside restores BBB integrity by preserving ZO-1 specifically at endothelial tight junctions. These effects are mediated by AMPK activation, as AMPK inhibition abolishes Gardenoside’s benefits both *in vitro* and *in vivo*. The compound protects HBMVECs against OGD/R injury in a dose-dependent manner. Gardenoside shows comparable efficacy in male and female mice, supporting its potential as a stroke therapy.

## Data Availability

The raw data supporting the conclusions of this article will be made available by the authors, without undue reservation.

## Supplementary material

SUPPLEMENTARY FIGURE S1 Dose–response curve for gardenoside in HBMVECs. HBMVECs were treated with increasing concentrations of gardenoside (0, 1, 5, 10, 20, 50 μM) for 24 h under normoxic conditions. Cell viability was assessed by CCK-8 assay. Data represent mean ± SD (*n* = 4 independent biological replicates). ^※^, ^※※^*p* < 0.05, 0.01 vs. control group.

## References

[ref1] Abbott N. J. Patabendige A. A. Dolman D. E. Yusof S. R. Begley D. J. (2010). Structure and function of the blood-brain barrier. Neurobiol. Dis. 37, 13–25. doi: 10.1016/j.nbd.2009.07.030 19664713

[ref2] Abdullahi W. Tripathi D. Ronaldson P. T. (2018). Blood-brain barrier dysfunction in ischemic stroke: targeting tight junctions and transporters for vascular protection. Am. J. Physiol. Cell Physiol. 315, C343–C356. doi: 10.1152/ajpcell.00095.2018, 29949404 PMC6171039

[ref3] Bederson J. B. Pitts L. H. Germano S. M. Nishimura M. C. Davis R. L. Bartkowski H. M. (1986). Evaluation of 2,3,5-triphenyltetrazolium chloride as a stain for detection and quantification of experimental cerebral infarction in rats. Stroke 17, 1304–1308. doi: 10.1161/01.str.17.6.1304, 2433817

[ref4] Benjamin E. J. Muntner P. Alonso A. Bittencourt M. S. Callaway C. W. Carson A. P. . (2019). Heart disease and stroke Statistics-2019 update: a report from the American Heart Association. Circulation 139, e56–e528. doi: 10.1161/CIR.0000000000000659, 30700139

[ref5] Bushnell C. LD M. C. Awad I. A. Chireau M. V. Fedder W. N. Furie K. L. . (2014). Guidelines for the prevention of stroke in women: a statement for healthcare professionals from the American Heart Association/American Stroke Association. Stroke 45, 1545–1588. doi: 10.1161/01.str.0000442009.06663.48, 24503673 PMC10152977

[ref6] Bustin S. A. Beaulieu J. F. Huggett J. Jaggi R. Kibenge F. S. Olsvik P. A. . (2010). MIQE précis: practical implementation of minimum standard guidelines for fluorescence-based quantitative real-time PCR experiments. BMC Mol. Biol. 11:74. doi: 10.1186/1471-2199-11-74, 20858237 PMC2955025

[ref7] Bustin S. A. Benes V. Garson J. A. Hellemans J. Huggett J. Kubista M. . (2009). The MIQE guidelines: minimum information for publication of quantitative real-time PCR experiments. Clin. Chem. 55, 611–622. doi: 10.1373/clinchem.2008.112797 19246619

[ref8] Daneman R. Prat A. (2015). The blood-brain barrier. Cold Spring Harb. Perspect. Biol. 7:a020412. doi: 10.1101/cshperspect.a020412, 25561720 PMC4292164

[ref9] Dirnagl U. (2006). Bench to bedside: the quest for quality in experimental stroke research. J. Cereb. Blood Flow Metab. 26, 1465–1478. doi: 10.1038/sj.jcbfm.9600298, 16525413

[ref10] Duan Y. Deng Y. Tang F. Li J. (2024). Lifibrate attenuates blood-brain barrier damage following ischemic stroke via the MLCK/p-MLC/ZO-1 axis. Aging (Albany NY) 16, 6135–6146. doi: 10.18632/aging.205692, 38546384 PMC11042934

[ref11] Engel O. Kolodziej S. Dirnagl U. Prinz V. (2011). Modeling stroke in mice - middle cerebral artery occlusion with the filament model. J. Vis. Exp. 47:2423. doi: 10.3791/2423, 21248698 PMC3182649

[ref12] Fisher M. Feuerstein G. Howells D. W. Hurn P. D. Kent T. A. Savitz S. I. . (2009). Update of the stroke therapy academic industry roundtable preclinical recommendations. Stroke 40, 2244–2250. doi: 10.1161/STROKEAHA.108.541128, 19246690 PMC2888275

[ref13] Gao H. M. Chen H. Cui G. Y. Hu J. X. (2023). Damage mechanism and therapy progress of the blood-brain barrier after ischemic stroke. Cell Biosci. 13:196. doi: 10.1186/s13578-023-01126-z, 37915036 PMC10619327

[ref14] GBD 2019 Stroke Collaborators (2021). Global, regional, and national burden of stroke and its risk factors, 1990–2019: a systematic analysis for the global burden of disease study 2019. Lancet Neurol. 20, 795–820. doi: 10.1016/S1474-4422(21)00252-0, 34487721 PMC8443449

[ref15] Hou Z. Sun L. Jiang Z. Zeng T. Wu P. Huang J. . (2024). Neuropharmacological insights into *Gardenia jasminoides* Ellis: harnessing therapeutic potential for central nervous system disorders. Phytomedicine 125:155374. doi: 10.1016/j.phymed.2024.155374, 38301302

[ref16] Iadecola C. Anrather J. (2011). The immunology of stroke: from mechanisms to translation. Nat. Med. 17, 796–808. doi: 10.1038/nm.2399, 21738161 PMC3137275

[ref17] Jia J. Cheng J. Ni J. Zhen X. (2015). Neuropharmacological actions of metformin in stroke. Curr. Neuropharmacol. 13, 389–394. doi: 10.2174/1570159x13666150205143555, 26411966 PMC4812800

[ref18] Jiang S. Li T. Ji T. Yi W. Yang Z. Wang S. . (2018). AMPK: potential therapeutic target for ischemic stroke. Theranostics 8, 4535–4551. doi: 10.7150/thno.25674, 30214637 PMC6134933

[ref19] Jiao H. Wang Z. Liu Y. Wang P. Xue Y. (2011). Specific role of tight junction proteins claudin-5, occludin, and ZO-1 of the blood-brain barrier in a focal cerebral ischemic insult. J. Mol. Neurosci. 44, 130–139. doi: 10.1007/s12031-011-9496-4, 21318404

[ref20] Jin R. Yang G. Li G. (2010). Inflammatory mechanisms in ischemic stroke: role of inflammatory cells. J. Leukoc. Biol. 87, 779–789. doi: 10.1189/jlb.1109766, 20130219 PMC2858674

[ref21] Jing L. Wang J. G. Zhang J. Z. Cao C. X. Chang Y. Dong J. D. . (2014). Upregulation of ICAM-1 in diabetic rats after transient forebrain ischemia and reperfusion injury. J. Inflamm. (Lond). 11:35. doi: 10.1186/s12950-014-0035-2, 25389378 PMC4226864

[ref22] Johnston S. C. Easton J. D. Farrant M. Barsan W. Conwit R. A. Elm J. J. . (2018). Clopidogrel and aspirin in acute ischemic stroke and high-risk TIA. N. Engl. J. Med. 379, 215–225. doi: 10.1056/NEJMoa1800410, 29766750 PMC6193486

[ref23] Kawata Y. Hattori M. Akao T. Kobashi K. Namba T. (1991). Formation of nitrogen-containing metabolites from geniposide and gardenoside by human intestinal bacteria. Planta Med. 57, 536–542. doi: 10.1055/s-2006-960201, 1818345

[ref24] Kim B. J. Hancock B. M. Bermudez A. Del Cid N. Reyes E. van Sorge N. M. . (2015). Bacterial induction of Snail1 contributes to blood-brain barrier disruption. J. Clin. Invest. 125, 2473–2483. doi: 10.1172/JCI74159, 25961453 PMC4497739

[ref25] Li H. B. Ma J. F. Mei Y. D. Liu L. X. Cao Z. Y. Shi D. F. . (2022). Two new iridoid glycosides from the fruit of *Gardenia jasminoides*. Nat. Prod. Res. 36, 186–192. doi: 10.1080/14786419.2020.1775227, 32594764

[ref26] Li J. McCullough L. D. (2010). Effects of AMP-activated protein kinase in cerebral ischemia. J. Cereb. Blood Flow Metab. 30, 480–492. doi: 10.1038/jcbfm.2009.255, 20010958 PMC2852687

[ref27] Liu J. Jin X. Liu K. J. Liu W. (2012). Matrix metalloproteinase-2-mediated occludin degradation and caveolin-1-mediated claudin-5 redistribution contribute to blood-brain barrier damage in early ischemic stroke stage. J. Neurosci. 32, 3044–3057. doi: 10.1523/JNEUROSCI.6409-11.2012, 22378877 PMC3339570

[ref28] Liu Y. Tang G. Li Y. Wang Y. Chen X. Gu X. . (2014). Metformin attenuates blood-brain barrier disruption in mice following middle cerebral artery occlusion. J. Neuroinflammation 11:177. doi: 10.1186/s12974-014-0177-4, 25315906 PMC4201919

[ref29] Liu F. Wang Y. Yao W. Xue Y. Zhou J. Liu Z. (2019). Geniposide attenuates neonatal mouse brain injury after hypoxic-ischemia involving the activation of PI3K/Akt signaling pathway. J. Chem. Neuroanat. 102:101687. doi: 10.1016/j.jchemneu.2019.101687, 31562918

[ref30] Longa E. Z. Weinstein P. R. Carlson S. Cummins R. (1989). Reversible middle cerebral artery occlusion without craniectomy in rats. Stroke 20, 84–91. doi: 10.1161/01.str.20.1.84, 2643202

[ref31] Mihaylova M. M. Shaw R. J. (2011). The AMPK signalling pathway coordinates cell growth, autophagy and metabolism. Nat. Cell Biol. 13, 1016–1023. doi: 10.1038/ncb2329, 21892142 PMC3249400

[ref32] Olivier S. Leclerc J. Grenier A. Foretz M. Tamburini J. Viollet B. (2019). AMPK activation promotes tight junction assembly in intestinal epithelial Caco-2 cells. Int. J. Mol. Sci. 20:5171. doi: 10.3390/ijms20205171, 31635305 PMC6829419

[ref33] Rousselet E. Kriz J. Seidah N. G. (2012). Mouse model of intraluminal MCAO: cerebral infarct evaluation by cresyl violet staining. J. Vis. Exp. 69:4038. doi: 10.3791/4038, 23168377 PMC3520579

[ref34] Salminen A. Hyttinen J. M. Kaarniranta K. (2011). AMP-activated protein kinase inhibits NF-κB signaling and inflammation: impact on healthspan and lifespan. J. Mol. Med. (Berl) 89, 667–676. doi: 10.1007/s00109-011-0748-0, 21431325 PMC3111671

[ref35] Salminen A. Kaarniranta K. Kauppinen A. (2016). AMPK and HIF signaling pathways regulate both longevity and cancer growth: the good news and the bad news about survival mechanisms. Biogerontology 17, 655–680. doi: 10.1007/s10522-016-9655-7, 27259535

[ref36] Sandoval K. E. Witt K. A. (2008). Blood-brain barrier tight junction permeability and ischemic stroke. Neurobiol. Dis. 32, 200–219. doi: 10.1016/j.nbd.2008.08.005 18790057

[ref37] Spandidos A. Wang X. Wang H. Seed B. (2010). PrimerBank: a resource of human and mouse PCR primer pairs for gene expression detection and quantification. Nucleic Acids Res. 38, D792–D799. doi: 10.1093/nar/gkp1005, 19906719 PMC2808898

[ref38] Stowe A. M. Adair-Kirk T. L. Gonzales E. R. Perez R. S. Shah A. R. Park T. S. . (2009). Neutrophil elastase and neurovascular injury following focal stroke and reperfusion. Neurobiol. Dis. 35, 82–90. doi: 10.1016/j.nbd.2009.04.006, 19393318 PMC2708673

[ref39] Stroke Therapy Academic Industry Roundtable (STAIR) (1999). Recommendations for standards regarding preclinical neuroprotective and restorative drug development. Stroke 30, 2752–2758. doi: 10.1161/01.str.30.12.2752, 10583007

[ref40] Sun Q. Zhang X. Fan J. Zhang L. Ji H. Xue J. . (2023). Geniposide protected against cerebral ischemic injury through the anti-inflammatory effect via the NF-κB signaling pathway. Transl. Neurosci. 14:20220273. doi: 10.1515/tnsci-2022-0273, 37333874 PMC10276575

[ref41] Tornavaca O. Chia M. Dufton N. Almagro L. O. Conway D. E. Randi A. M. . (2015). ZO-1 controls endothelial adherens junctions, cell-cell tension, angiogenesis, and barrier formation. J. Cell Biol. 208, 821–838. doi: 10.1083/jcb.201404140, 25753039 PMC4362456

[ref42] Vemuganti R. Dempsey R. J. Bowen K. K. (2004). Inhibition of intercellular adhesion molecule-1 protein expression by antisense oligonucleotides is neuroprotective after transient middle cerebral artery occlusion in rat. Stroke 35, 179–184. doi: 10.1161/01.STR.0000106479.53235.3E, 14657453

[ref43] Wang J. Hou J. Zhang P. Li D. Zhang C. Liu J. (2012). Geniposide reduces inflammatory responses of oxygen-glucose deprived rat microglial cells via inhibition of the TLR4 signaling pathway. Neurochem. Res. 37, 2235–2248. doi: 10.1007/s11064-012-0852-8, 22869019

[ref44] Wang X. Seed B. (2003). A PCR primer bank for quantitative gene expression analysis. Nucleic Acids Res. 31:e154. doi: 10.1093/nar/gng154, 14654707 PMC291882

[ref45] Wu S. Zou M. H. (2020). AMPK, mitochondrial function, and cardiovascular disease. Int. J. Mol. Sci. 21:4987. doi: 10.3390/ijms21144987, 32679729 PMC7404275

[ref46] Xie W. Zeng Y. Zheng Y. Cai B. (2023). Activated AMPK protects against chronic cerebral ischemia in bilateral carotid artery stenosis mice. Cell. Mol. Neurobiol. 43, 2325–2335. doi: 10.1007/s10571-022-01312-6, 36441266 PMC11412192

[ref47] Yang Y. Rosenberg G. A. (2011). Blood-brain barrier breakdown in acute and chronic cerebrovascular disease. Stroke 42, 3323–3328. doi: 10.1161/STROKEAHA.110.608257, 21940972 PMC3584169

[ref48] Yu H. Liu Q. Chen G. Huang L. Luo M. Lv D. . (2022). SIRT3-AMPK signaling pathway as a protective target in endothelial dysfunction of early sepsis. Int. Immunopharmacol. 106:108600. doi: 10.1016/j.intimp.2022.108600, 35217431

[ref49] Zhang R. L. Chopp M. Zaloga C. Zhang Z. G. Jiang N. Gautam S. C. . (1995). The temporal profiles of ICAM-1 protein and mRNA expression after transient MCA occlusion in the rat. Brain Res. 682, 182–188. doi: 10.1016/0006-8993(95)00346-r, 7552309

[ref50] Zhang H. Lai Q. Li Y. Liu Y. Yang M. (2017). Learning and memory improvement and neuroprotection of *Gardenia jasminoides* (fructus gardenia) extract on ischemic brain injury rats. J. Ethnopharmacol. 196, 225–235. doi: 10.1016/j.jep.2016.11.042, 27940085

[ref51] Zhao Z. Hu J. Gao X. Liang H. Liu Z. (2014). Activation of AMPK attenuates lipopolysaccharide-impaired integrity and function of blood-brain barrier in human brain microvascular endothelial cells. Exp. Mol. Pathol. 97, 386–392. doi: 10.1016/j.yexmp.2014.09.006, 25220346

[ref52] Zhou Y. X. Zhang R. Q. Rahman K. Cao Z. X. Zhang H. Peng C. (2019). Diverse pharmacological activities and potential medicinal benefits of geniposide. Evid. Based Complement. Alternat. Med. 2019, 1–15. doi: 10.1155/2019/4925682, 31118959 PMC6500620

