# Supplementary figures and images for: Gardenoside restores blood–brain barrier integrity following ischemic stroke via AMPK-dependent ZO-1 preservation
Source: Front Behav Neurosci. 2026 May 12;20:1768582. doi: 10.3389/fnbeh.2026.1768582 (PMC13201230; doi:10.3389/fnbeh.2026.1768582)

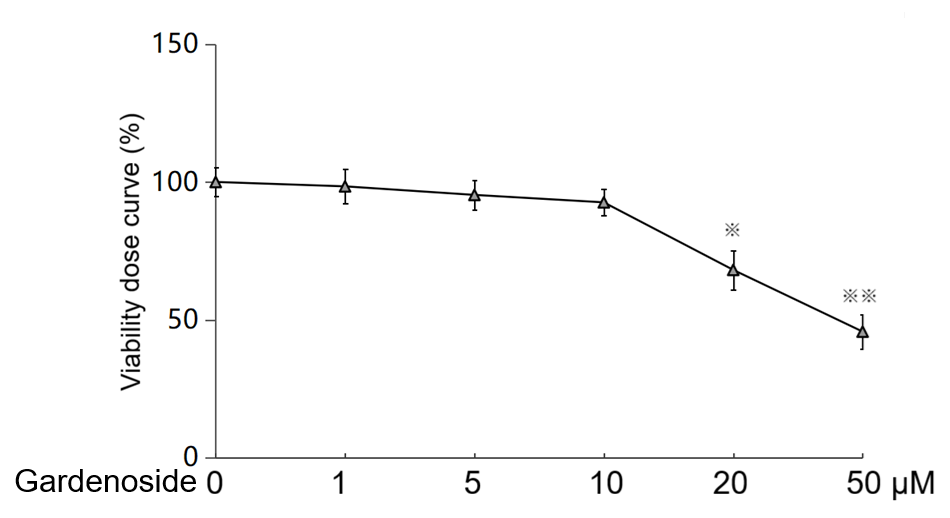

Supplement: SUPPLEMENTARY FIGURE S1 — Dose–response curve for gardenoside in HBMVECs. HBMVECs were treated with increasing concentrations of gardenoside (0, 1, 5, 10, 20, 50 μM) for 24 h under normoxic conditions. Cell viability was assessed by CCK-8 assay. Data represent mean ± SD (n = 4 independent biological replicates). ※, ※※p < 0.05, 0.01 vs. control group. [file Image_1.tif]
